# Supplementary material for: Tumors defective in homologous recombination rely on oxidative metabolism: relevance to treatments with PARP inhibitors
Source: EMBO Mol Med. 2020 May 13;12(6):e11217. doi: 10.15252/emmm.201911217 (PMC7278557; doi:10.15252/emmm.201911217)
Supplement: Supplementary file 1 — Appendix [file EMMM-12-e11217-s001.pdf]

Tumors defective in homologous recombination rely on oxidative metabolism: Relevance to treatments with PARP inhibitors

Álvaro Lahiguera, Petra Hyroššová, Agnès Figueras, Diana Garzón, Roger Moreno, Vanessa Soto-Cerrato, Iain McNeish, Violeta Serra, Conxi Lazaro, Pilar Barretina, Joan Brunet, Javier Menéndez, Xavier Matias-Guiu, August Vidal, Alberto Villanueva, Barbie Taylor-Harding, Hisashi Tanaka, Sandra Orsulic, Alexandra Junz, Oscar Yanes, Cristina Muñoz-Pinedo, Luís Palomero, Miquel Àngel Pujana, José Carlos Perales & Francesc Viñals

APPENDIX FILE

Table of Content

- Appendix Figure S1
- Appendix Figure S2
- Appendix Figure S3
- Appendix Figure S4
- Appendix Figure S5
- Appendix Figure S6
- Appendix Figure S7
- Appendix Figure S8
- Appendix Figure Legends

Appendix Figure S1

A)

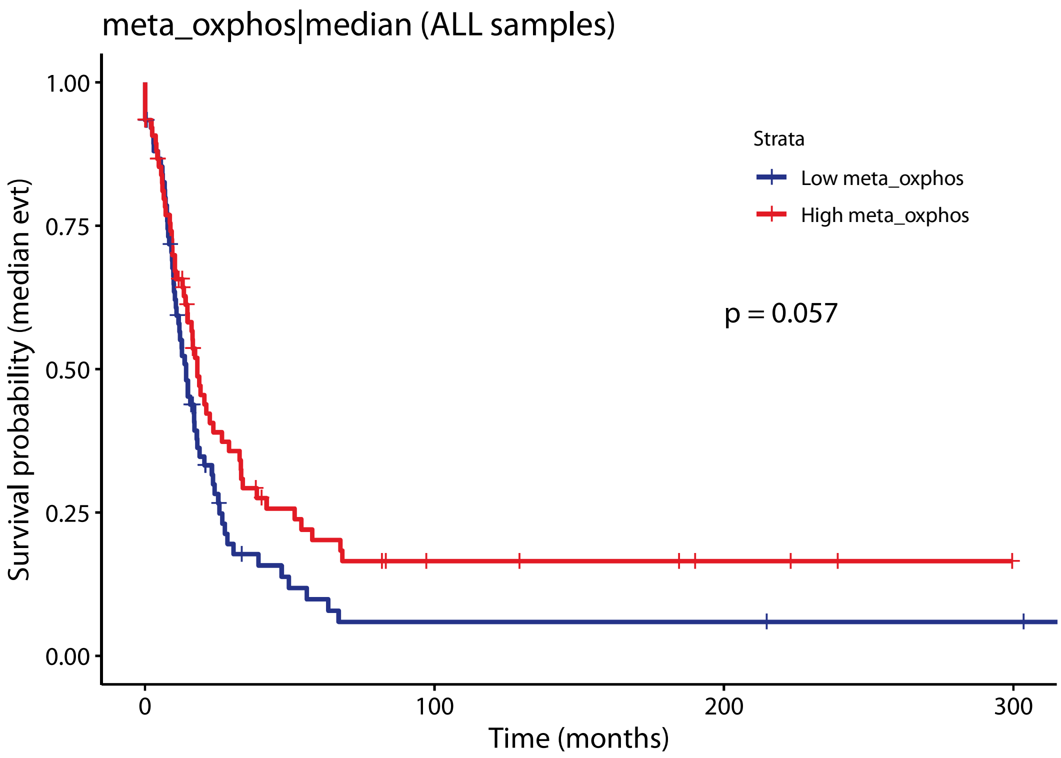

B)

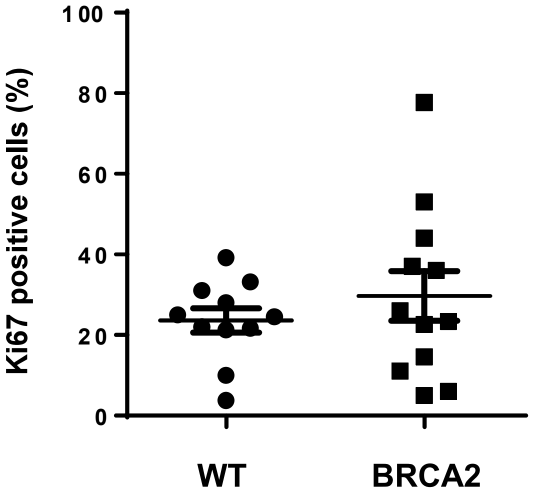

Appendix Figure S2

A)

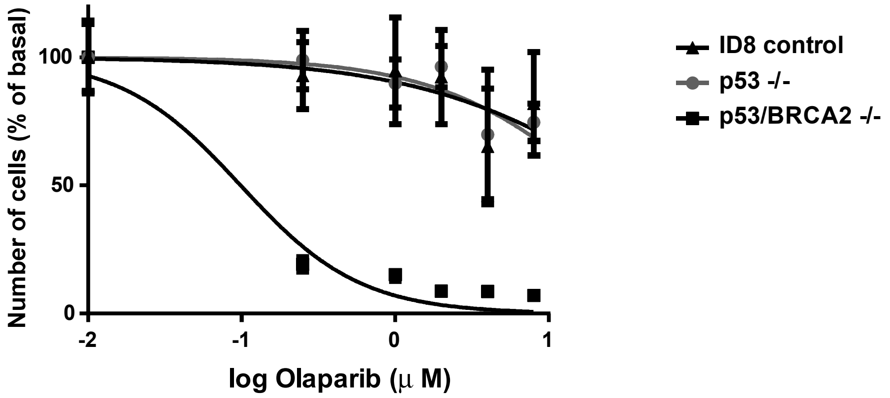

B)

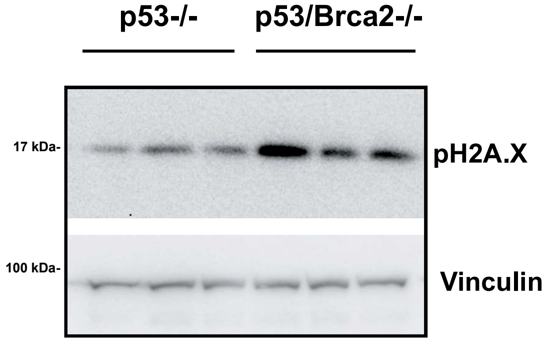

Appendix Figure S3

A) SKOV-3-R cells

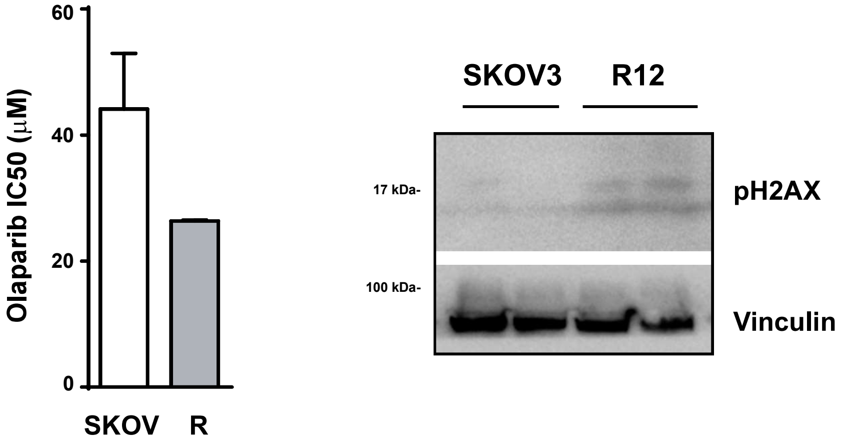

B) Ovarian cancer *Trp53 -/- or Trp53/Brca1 -/-* , *Akt, Myc*

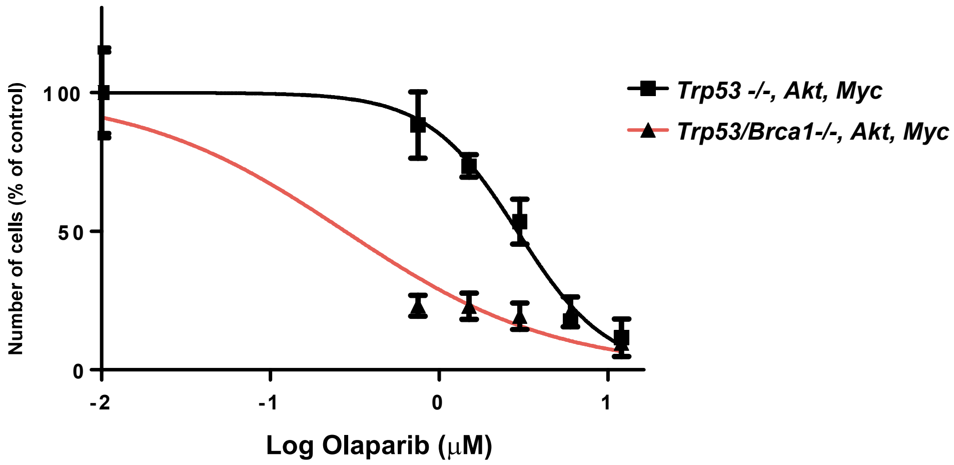

C) MEFs *Trp53-/-* or *Trp53/TSC2 -/-*

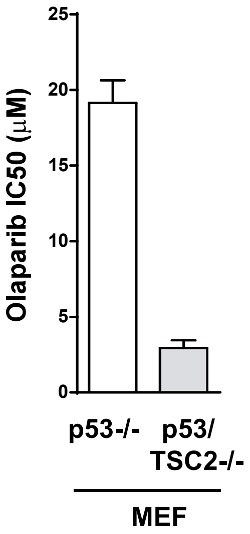

Appendix Figure S4

A)

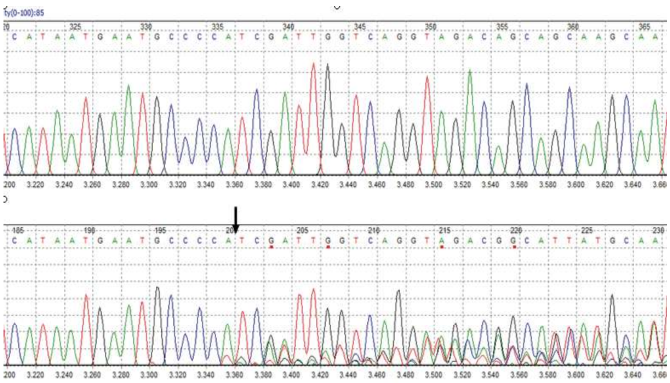

B)

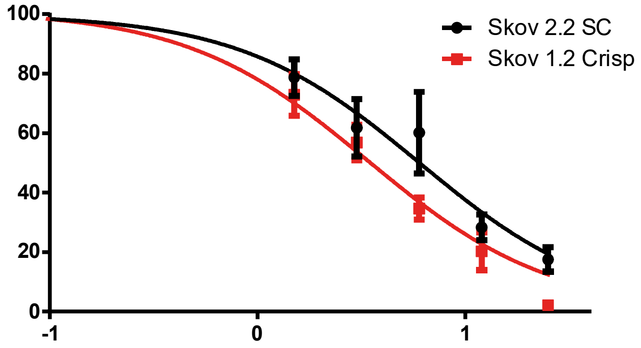

C)

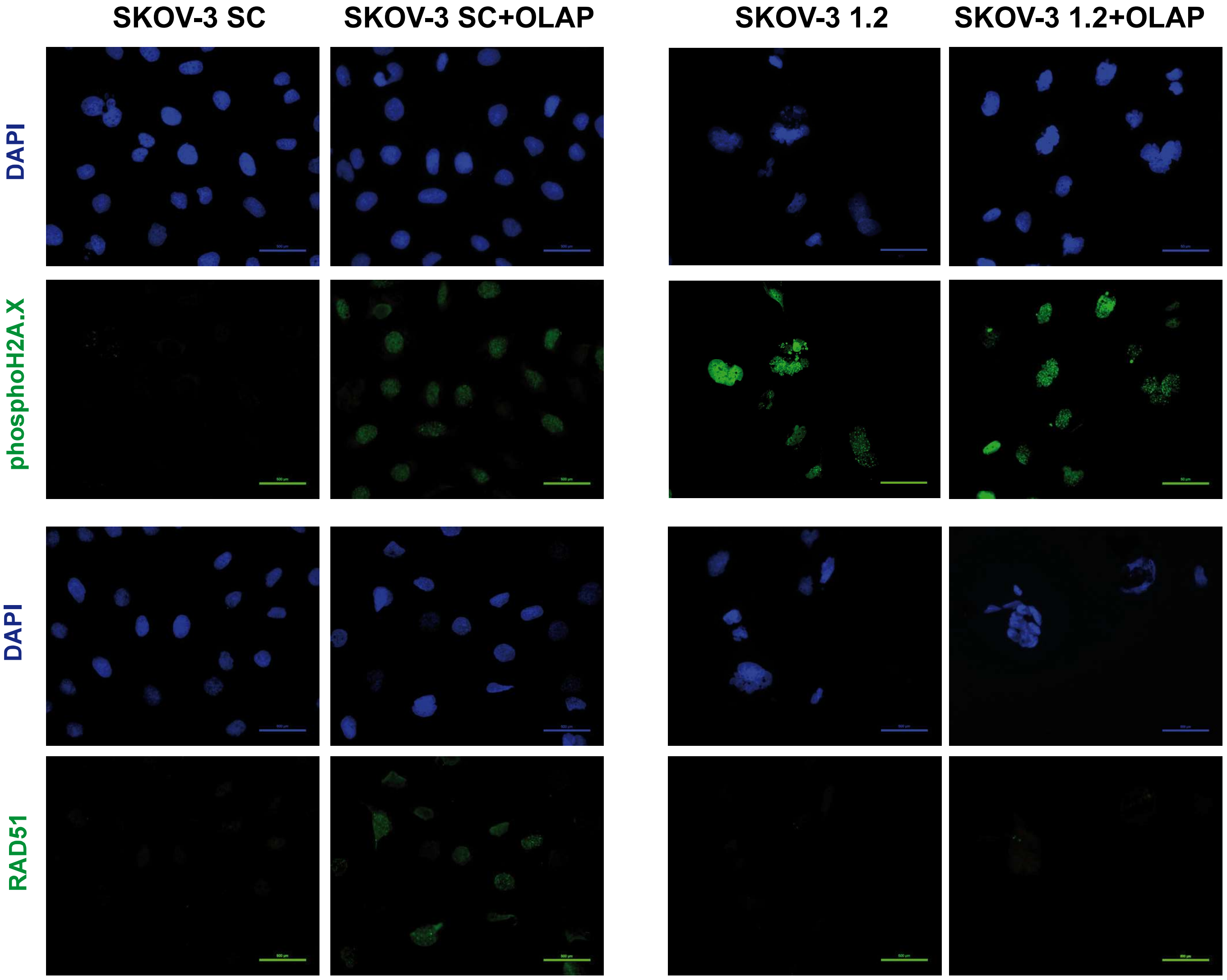

Appendix Figure S5

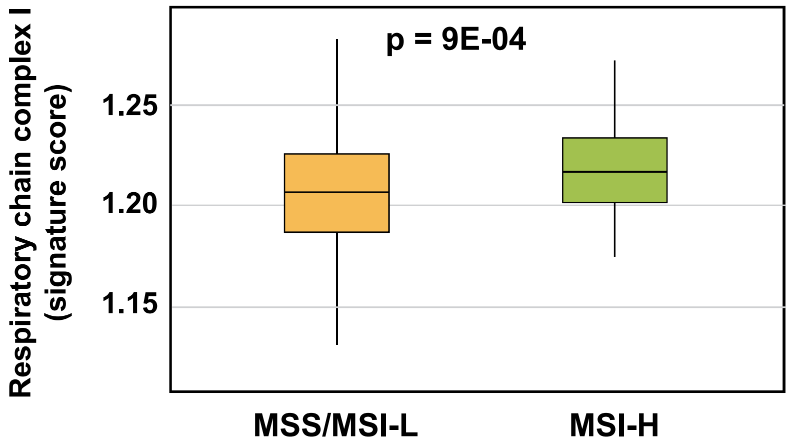

Appendix Figure S6

A)

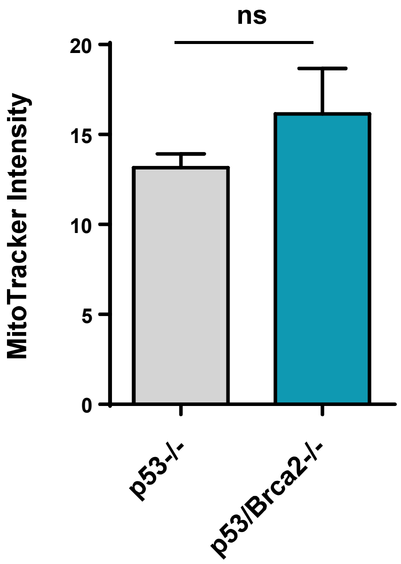

B)

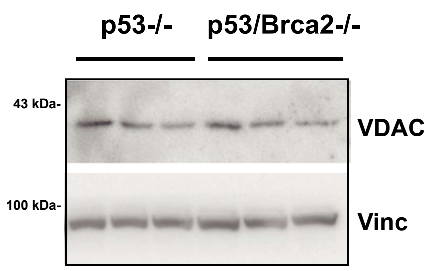

Appendix Figure S7

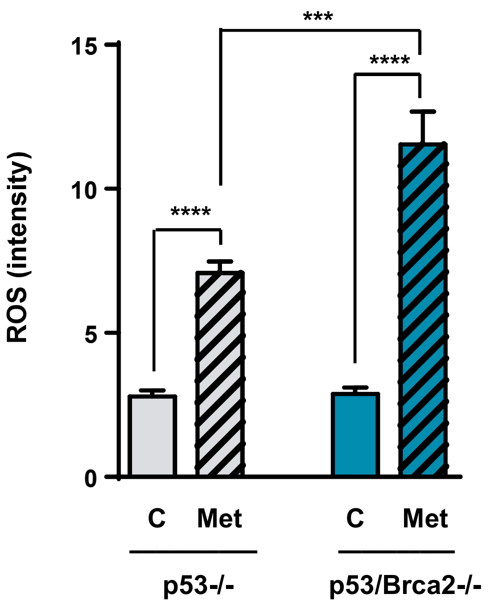

Appendix Figure S8

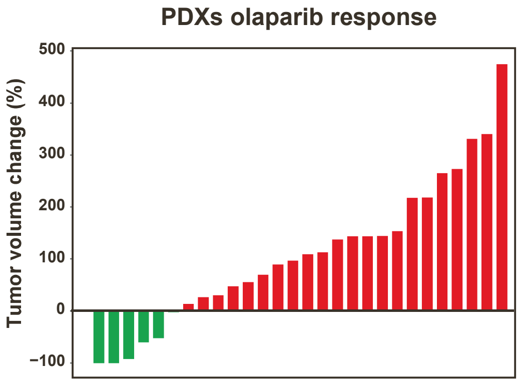

APPENDIX FIGURE LEGENDS

Appendix Figure S1. Association between OXPHOS gene signature in ovarian tumors and clinical outcome.

(A) High or low OXPHOS ssGSEA signature expression was analyzed in ovarian cancer patients as described in Materials and Methods and correlated with clinical outcome. Multivariate Cox regression analyses using the survfit and survminer packages in R software were applied.

(B) % of Ki67 positive tumoral cells in tumor tissue sections from wild-type and *BRCA2*-mutated high-grade serous tumors. Error bars indicate the SEM.

Appendix Figure S2. Olaparib sensitivity and DNA damage in *Trp53*-deleted and *Trp53/Brca2*-deleted ovarian tumor cells.

(A) ID8 control, *Trp53*-deleted and *Trp53/Brca2*-deleted ovarian tumor cells were incubated for 3 days over a range of olaparib concentrations. Cell viability was measured as the frequency of cells stained with crystal violet. Results are expressed relative to a zero level of olaparib. Each data-point represents the mean and SEM of eight independent determinations.

(B) Expression of phosphorylated histone H2A.X (Serine 139) and vinculin in ID8 *Trp53*-deleted (n=3) and *Trp53/Brca2*-deleted (n=3) ovarian tumor cells.

Appendix Figure S3. Olaparib sensitivity and DNA damage in different cell lines.

(A) Left panel: SKOV-3 and SKOV-3-R human ovarian tumor cells were incubated for 3 days over a range of olaparib concentrations. Cell viability was measured as the frequency of cells stained with crystal violet. The IC_{50} was determined by representing results relative to the control. Each data-point represents the mean and SEM of three independent determinations.

Right panel: Expression of phosphorylated histone H2A.X (Serine 139) and vinculin in SKOV-3 (n=2) or SKOV-3-R (n=2) ovarian tumor cells analyzed by western blot. A representative blot is shown.

(B) *Trp53*-/-, *myc* and *Akt* or *Trp53*-/-, *Brca1*-/-, *myc* and *Akt* ovarian mouse cancer cells were incubated for 3 days over a range of olaparib concentrations. Cell viability was measured as the frequency of cells stained with crystal violet. The IC_{50} was determined by representing results relative to the control. Each data-point represents the mean and SEM of three independent determinations.

(C) *Trp53*-deficient and double *Trp53*/*Tsc2*-deleted MEFs were incubated for 3 days over a range of olaparib concentrations. Cell viability was measured as the frequency of cells stained with crystal violet. The IC_{50} was determined by representing results relative to the control. Each data-point represents the mean and SEM of three independent determinations.

Appendix Figure S4. Olaparib sensitivity and DNA damage in CRISPR-CAS9 *BRCA2*-deleted SKOV-3 cells.

A) Sequencing data from the BRCA2 gene fragment targeted by the GenCRISPR™ gRNA/Cas9 lentiCRISPR v2 vector in the 1.2 cells (bottom

panel). The wild-type (WT) sequence is also shown (top panel). The arrow indicates the start of the CRISPR-CAS9 targeting sequence in the BRCA2 gene.

B) Control (2.2 SC) and *BRCA2*-deleted SKOV-3 (1.2 Crisp) human ovarian tumor cells were incubated for 3 days over a range of olaparib concentrations. Cell viability was measured as the frequency of cells revealed by violet crystal staining. Results are expressed relative to the control in the absence of olaparib. Each data-point represents the mean and SEM of eight independent determinations.

C) Representative images of control (SC) and *BRCA2*-deleted SKOV-3 (1.2) human ovarian tumor cells, treated (right images) or not (left images) for 12 h with 5 μM olaparib, and immunostained with DAPI, phosphorylated Histone H2A.X (Ser 139) or RAD51. Bar 50 μm.

Appendix Figure S5. OXPHOS signature is associated to high genomic instability.

Box plot showing higher expression of a signature corresponding to the mitochondrial respiratory chain complex I in cancer cells lines classified as MSI-H (high genomic instability) relative to MSS/MSI-L (low genomic instability). The p value of the t-test is shown.

Appendix Figure S6. HR defects does not affect mitochondrial mass.

(A) ID8 *Trp53*-deleted and *Trp53/Brca2*-deleted ovarian tumor cells were incubated with green MitoTracker 30 minutes, cells were fixed and staining was evaluated by flow cytometry. The mean of three independent experiments is shown.

(B) VDAC mitochondrial marker and vinculin (as a loading control) expression were measured by immunoblotting in ID8 *Trp53*-deleted (n=3) and ID8 *Trp53/Brca2*-deleted (n=3) ovarian tumor cells

Appendix Figure S7. HR defects do not affect mitochondrial ROS.

ID8 *Trp53*-deleted (n=7) and *Trp53/Brca2*-deleted (n=7) ovarian tumor cells were incubated for 2 days with complete DMEM medium in the absence or presence of 5 mM metformin. After this time, mitochondrial ROS were measured as described in the Materials and Methods. Each data-point represents the mean and SEM. Statistical significance of one-tailed Anova tests, Tukey's multiple comparisons test: ***, p<0.001.

Appendix Figure S8. Olaparib sensitivity of *BRCA1*-mutated PDX.

Graph showing tumor volume change after olaparib treatment in *BRCA1*-mutated breast cancer PDXs. These cases were subsequently classified as olaparib-sensitive (green, n=5) or olaparib-resistant (red, n=22).
