# Supplementary material for: Tumors defective in homologous recombination rely on oxidative metabolism: relevance to treatments with PARP inhibitors
Source: EMBO Mol Med. 2020 May 13;12(6):e11217. doi: 10.15252/emmm.201911217 (PMC7278557; doi:10.15252/emmm.201911217)
Supplement: Supplementary file 2 — Expanded View Figures PDF [file EMMM-12-e11217-s002.pdf]

Expanded View Figures

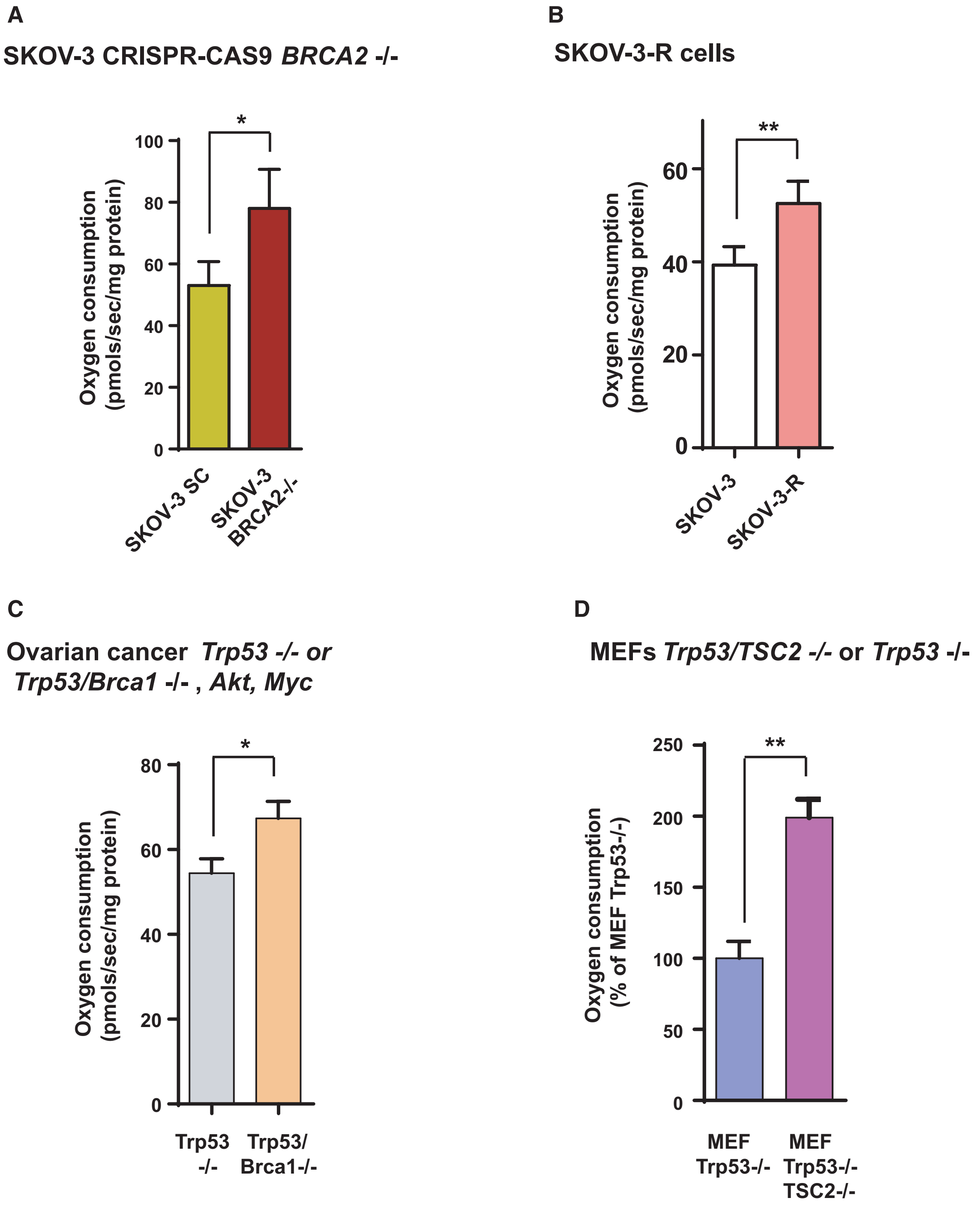

Figure EV1.

 © 2020 The Authors

 © 2020 The Authors

Figure EV1. HRD cell models show high OXPHOS.

A Oxygen consumption rates measured using an Oxygraph-2K in control SKOV-3 SC (n = 5) and SKOV-3-*BRCA2*$^{-/-}$ 1.2 (n = 3) human ovarian tumor cells. The bars indicate the mean and standard error (SEM). Statistical significance of two-tailed Mann–Whitney *U*-tests: *P = 0.0238.

B Oxygen consumption rates measured using an Oxygraph-2K in SKOV-3 control (n = 5) and SKOV-3-R (n = 5) human ovarian tumor cells. The bars indicate the mean and standard error (SEM). Statistical significance of two-tailed unpaired Mann–Whitney *U*-tests: **P = 0.007.

C Oxygen consumption rates measured using an Oxygraph-2K in *Trp53*$^{-/-}$, *myc* and *Akt* (n = 8) or *Trp53*$^{-/-}$, *Brca1*$^{-/-}$, *myc*, and *Akt* (n = 8) ovarian mouse cancer cells. The bars indicate the mean and standard error (SEM). Statistical significance of two-tailed Mann–Whitney *U*-tests: *P = 0.0281.

D Oxygen consumption rates measured using an Oxygraph-2K in *Trp53*-deficient (n = 4) and double *Trp53*/*Tsc2*-deleted MEFs (n = 4). The bars indicate the mean and standard error (SEM). Statistical significance of two-tailed Mann–Whitney *U*-tests: **P = 0.0014.

Figure EV2. HRD cell models show high OXPHOS and decreased glycolytic flux.

A Glucose consumption (mmol) was measured in DMEM from ID8 *Trp53*-deleted (n = 3, 0.11, 0.11, and 0.12) and *Trp53*/*Brca2*-deleted (n = 3, 0.10, 0.10, and 0.11) ovarian tumor cells grown for 24 h. Data were normalized with respect to protein content. Error bars indicate the SEM.

B Bars show quantification of *Hk2* and *LdhA* mRNA levels in ID8 *Trp53*-deleted (n = 4) and *Trp53*/*Brca2*-deleted (n = 6) ovarian tumor cells, normalized with respect to *Actin* gene. Error bars indicate the SEM. Statistical significance of two-tailed unpaired Mann–Whitney *U*-tests: *LdhA* (*P = 0.0381) and *Hk2* (**P = 0.0095).

C *Trp53*$^{-/-}$, *myc*, and *Akt* (n = 3) or *Trp53*$^{-/-}$, *Brca1*$^{-/-}$, *myc*, and *Akt* (n = 3) ovarian mouse cancer cells were grown in complete DMEM for 3 days. Cells were lysed and immunoblotted using the indicated antibodies.

D *Trp53*-deficient (n = 3) and double *Trp53*/*Tsc2*-deleted MEFs (n = 3) were grown in complete DMEM for 3 days. Cells were lysed and immunoblotted using the indicated antibodies.

E Oxygen consumption rates measured using an Oxygraph-2K in ID8 *Trp53*-deleted and ID8 *Trp53*/*Brca2*-deleted ovarian tumor cells grown for 2 days in basal Hank's medium (Basal), Hank's medium with 1 mM palmitoleic acid (FFAA), with 25 mM glucose (Gluco) or with 2 mM glutamine (Gln). Each data point represents the mean of two independent determinations.

Source data are available online for this figure.

© 2020 The Authors

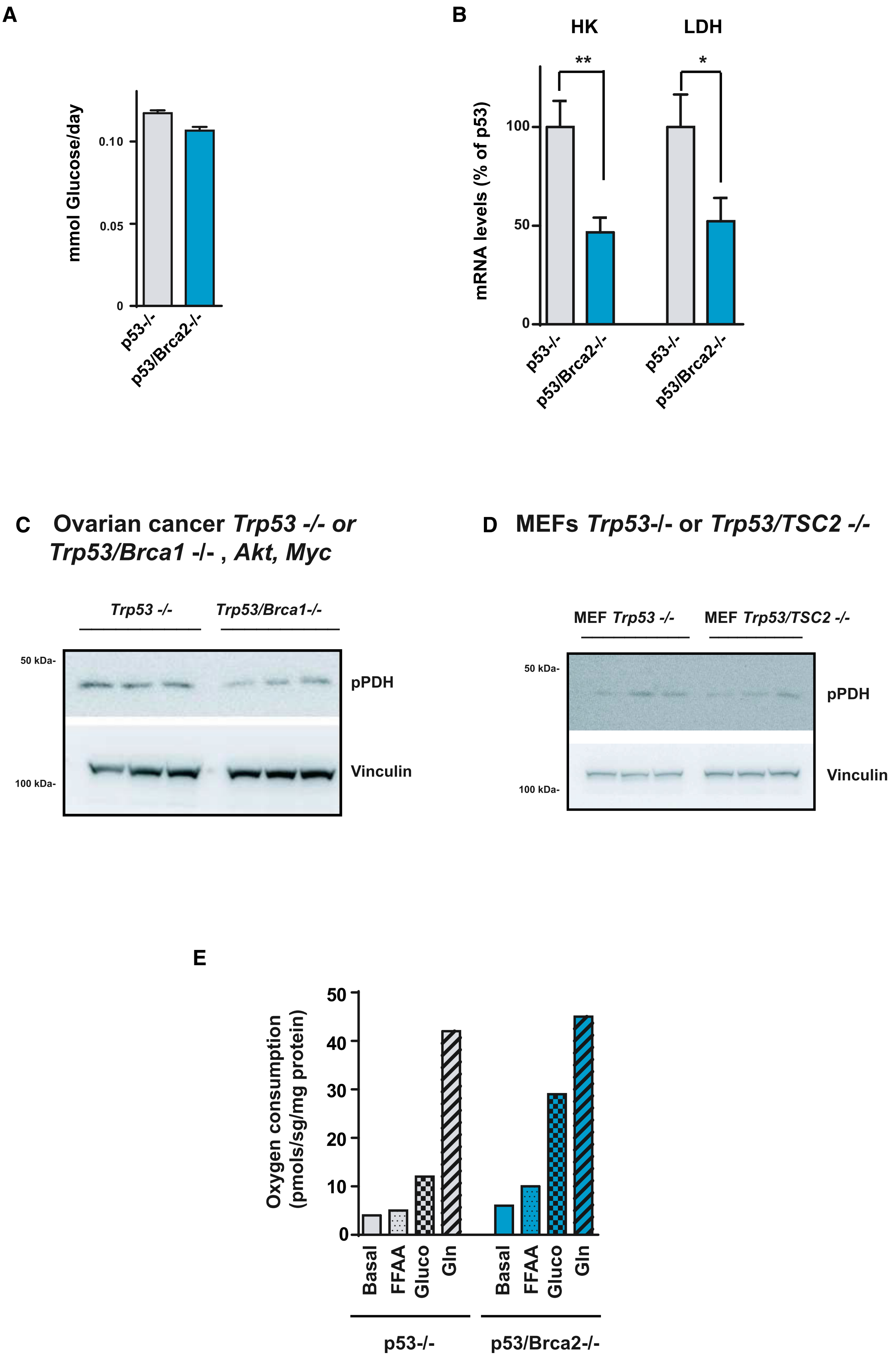

Figure EV2.

© 2020 The Authors

Figure EV3. Metformin does not affect apoptosis but induces cell cycle arrest.

A ID8 *Trp53*/*Brca2*-deleted ovarian tumor cells were incubated for 2 days with complete DMEM in the absence or presence of 2 mM metformin or 5 μM olaparib. After this time, cells were incubated with propidium iodide, fixed, and the cell cycle phase was evaluated as indicated. The mean of two independent experiments is shown.

B ID8 *Trp53*-deleted and *Trp53*/*Brca2*-deleted ovarian tumor cells were incubated for 2 days with complete DMEM in the absence or presence of 2 mM metformin or 5 μM olaparib. After this time, cells were fixed and Annexin-V-positive cells were evaluated as indicated. The mean and standard error (SEM) of two independent experiments is shown.

© 2020 The Authors

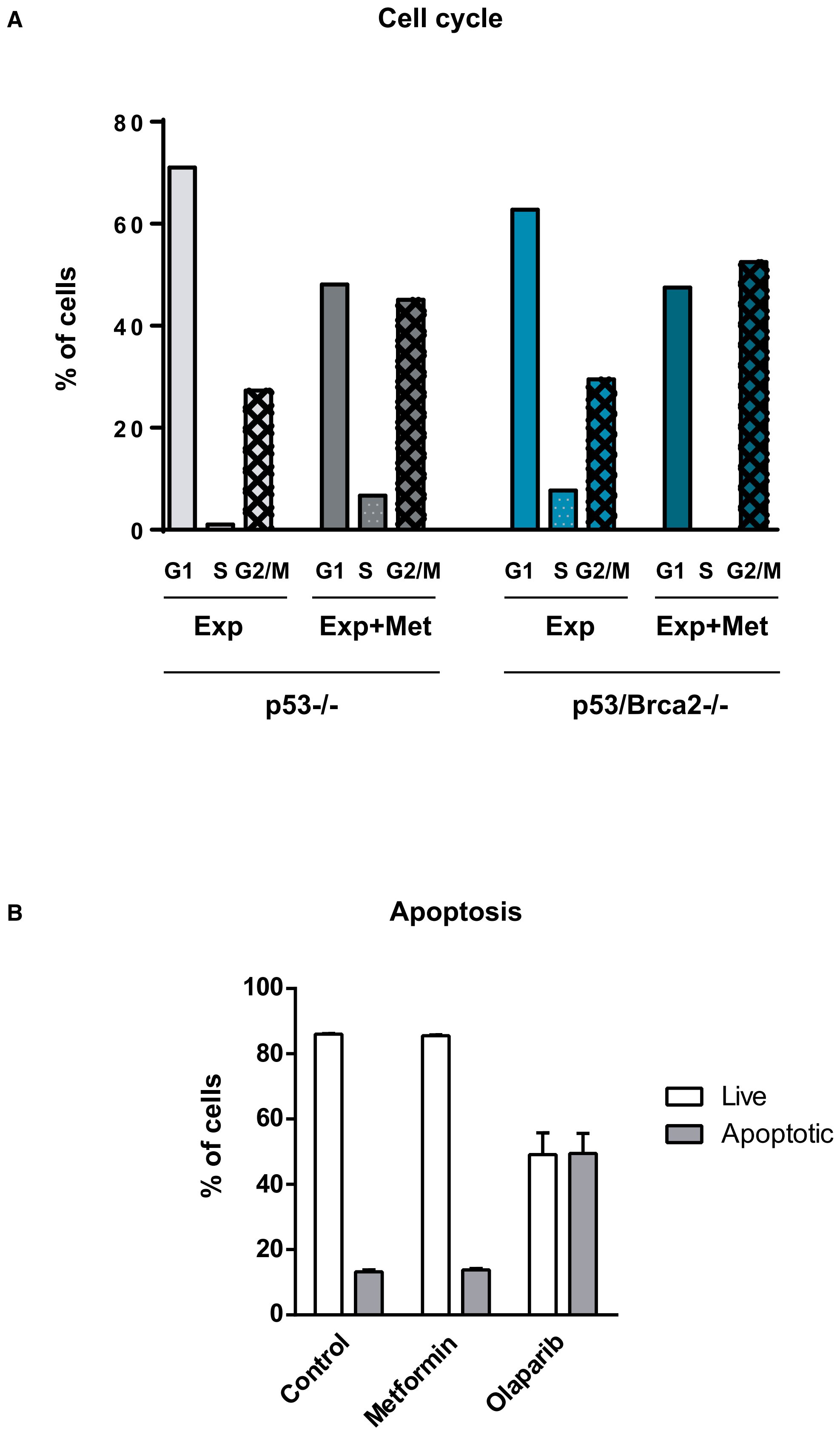

Figure EV3.

© 2020 The Authors

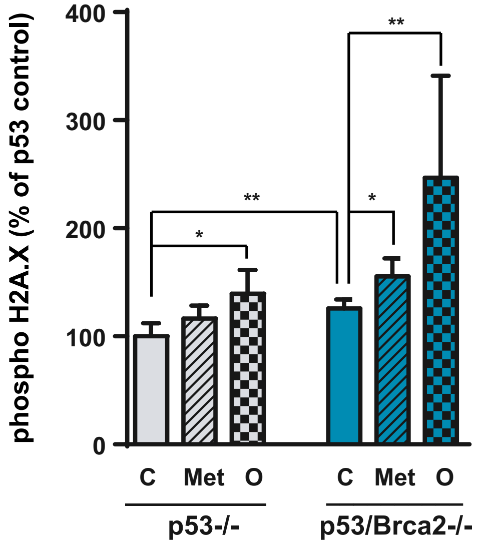

Figure EV4. Metformin increases DNA damage (phospho-histone H2A.X).
Phospho-histone H2A.X intensity was measured by flow cytometry in ID8 *Trp53*-deleted and *Trp53/Brca2*-deleted ovarian tumor cells incubated for 48 h with complete DMEM in the absence (C) or presence of 5 mM metformin (Met) or for the last 12 h in the presence of 5 μM olaparib (O). Each data point represents the mean of six independent determinations. Statistical significance of two-tailed unpaired Mann–Whitney *U*-tests: *Trp53*-deleted versus *Trp53/Brca2*-deleted in control conditions (**P = 0.0087); *Trp53*-deleted control versus olaparib-treated cells (*P = 0.019); *Trp53/Brca2*-deleted control compared with metformin-treated cells (*P = 0.0152); and *Trp53/Brca2*-deleted control compared with olaparib-treated cells (**P = 0.0022).

© 2020 The Authors

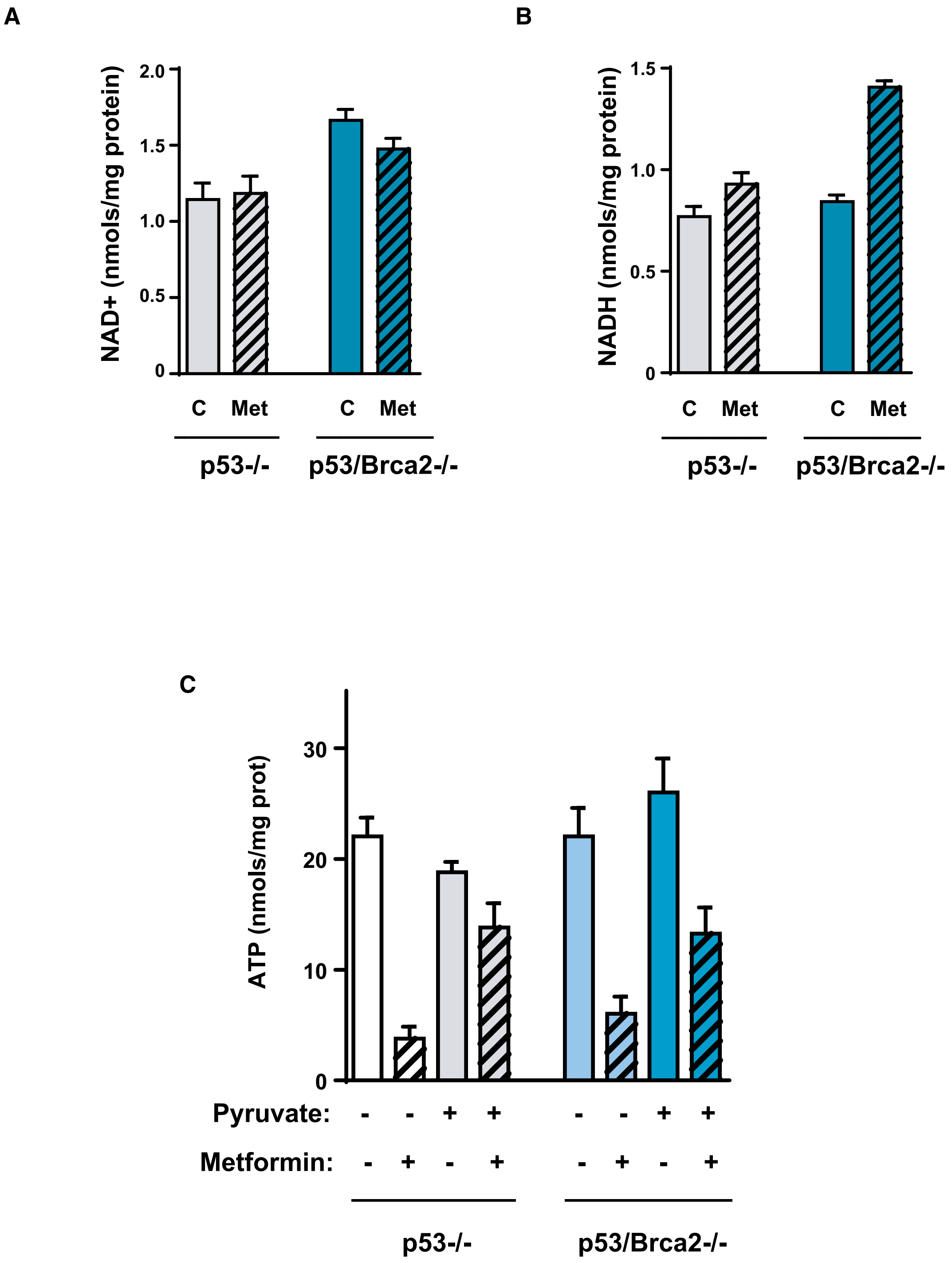

Figure EV5. Effect of metformin on NAD^+, NADH, and ATP levels.

A ID8 *Trp53*-deleted and *Trp53/Brca2*-deleted ovarian tumor cells were incubated for 48 h with complete DMEM in the absence or presence of 5 mM metformin. Cells were then lysed and total NAD^+ levels measured as indicated. Data were normalized with respect to protein content. The mean of four independent experiments is shown. Error bars indicate the SEM.

B Using the same extracts as in (A), total NADH levels were measured as indicated. Data were normalized with respect to protein content. The mean of four independent experiments is shown. Error bars indicate the SEM.

C ID8 *Trp53*-deleted (n = 4) and *Trp53/Brca2*-deleted (n = 4) ovarian tumor cells grown for 2 days in normal DMEM in the absence or presence of 2 mM metformin and in the absence or presence of 1 mM pyruvate. Cells were then lysed and total ATP levels measured as indicated. Data were normalized with respect to protein content. Error bars indicate the SEM.

© 2020 The Authors
