# Supplementary material for: Tumors defective in homologous recombination rely on oxidative metabolism: relevance to treatments with PARP inhibitors
Source: EMBO Mol Med. 2020 May 13;12(6):e11217. doi: 10.15252/emmm.201911217 (PMC7278557; doi:10.15252/emmm.201911217)
Supplement: Supplementary file 3 — Table EV1 [file EMMM-12-e11217-s003.pdf]

Table EV1. KEGG gene sets associated (FDR < 5%) with mutational signature 3 in ovarian and breast TCGA expression data.

**TCGA ovarian cancer**						
**Rank**	**KEGG pathway**	**Genes (n)**	**Enrichment score**	**Normalized ES**	**Nominal p value**	**FDR**
1	KEGG_PARKINSONS_DISEASE	110	-0,51	-2,38	0	0,00E+00
2	KEGG_OXIDATIVE_PHOSPHORYLATION	114	-0,41	-1,94	0	2,60E-02
3	KEGG_PORPHYRIN_AND_CHLOROPHYLL_METABOLISM	23	-0,57	-1,91	0	2,30E-02
4	KEGG_ALZHEIMERS_DISEASE	152	-0,38	-1,87	0	2,60E-02
5	KEGG_HUNTINGTONS_DISEASE	165	-0,37	-1,84	0	2,70E-02
**TCGA breast cancer**						
1	KEGG_PROTEASOME	43	-0,64	-2,59	0	0,00E+00
2	KEGG_PROTEIN_EXPORT	22	-0,67	-2,26	0	0,00E+00
3	KEGG_CELL_CYCLE	124	-0,46	-2,23	0	0,00E+00
4	KEGG_OOCYTE_MEIOSIS	110	-0,44	-2,15	0	0,00E+00
5	KEGG_UBIQUITIN_MEDIATED_PROTEOLYSIS	131	-0,43	-2,08	0	2,00E-03
6	KEGG_MISMATCH_REPAIR	23	-0,61	-2,06	0	3,00E-03
7	KEGG_ANTIGEN_PROCESSING_AND_PRESENTATION	61	-0,45	-1,93	0	8,00E-03
8	KEGG_PANCREATIC_CANCER	69	-0,44	-1,92	0	7,00E-03
9	KEGG_NUCLEOTIDE_EXCISION_REPAIR	44	-0,49	-1,91	0	8,00E-03
10	KEGG_TYPE_I_DIABETES_MELLITUS	23	-0,55	-1,88	0,005	1,10E-02
11	KEGG_PROPANOATE_METABOLISM	32	-0,52	-1,86	0	1,20E-02
12	KEGG_GRAFT_VERSUS_HOST_DISEASE	19	-0,57	-1,86	0	1,20E-02
13	KEGG_CITRATE_CYCLE_TCA_CYCLE	30	-0,51	-1,86	0,003	1,10E-02
14	KEGG_DNA_REPLICATION	36	-0,49	-1,85	0	1,10E-02
15	KEGG_COLORECTAL_CANCER	62	-0,42	-1,82	0	1,40E-02
16	KEGG_RNA_DEGRADATION	56	-0,42	-1,79	0	1,80E-02
17	KEGG_BASAL_TRANSCRIPTION_FACTORS	35	-0,47	-1,78	0	1,80E-02
18	KEGG_AMINOACYL_TRNA_BIOSYNTHESIS	41	-0,44	-1,75	0,006	2,10E-02
19	KEGG_VALINE_LEUCINE_AND_ISOLEUCINE_DEGRADATION	44	-0,44	-1,75	0,003	2,10E-02
20	KEGG_PROGESTERONE_MEDIATED_OOCYTE_MATURATION	85	-0,37	-1,69	0	3,20E-02
21	KEGG_B_CELL_RECEPTOR_SIGNALING_PATHWAY	74	-0,38	-1,66	0,003	3,80E-02
22	KEGG_SYSTEMIC_LUPUS_ERYTHEMATOSUS	119	-0,34	-1,64	0	4,30E-02
