# Supplementary figures and images for: Tumors defective in homologous recombination rely on oxidative metabolism: relevance to treatments with PARP inhibitors
Source: EMBO Mol Med. 2020 May 13;12(6):e11217. doi: 10.15252/emmm.201911217 (PMC7278557; doi:10.15252/emmm.201911217)

Fig. EV2

C)

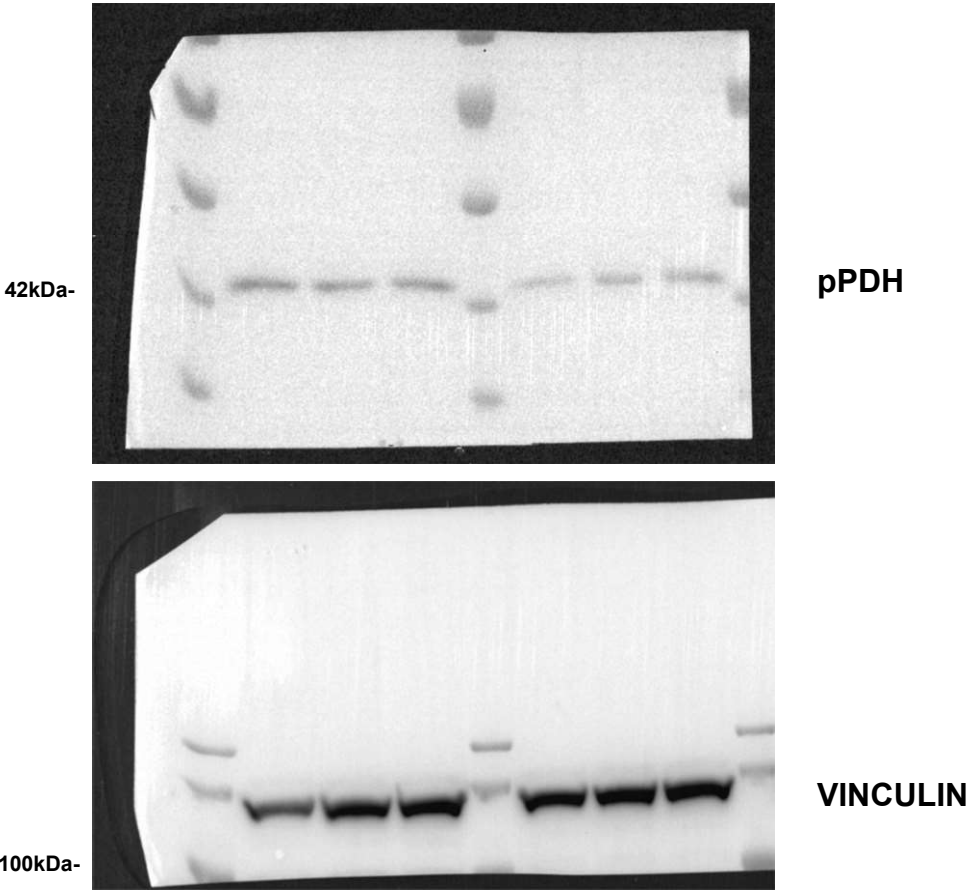

D)

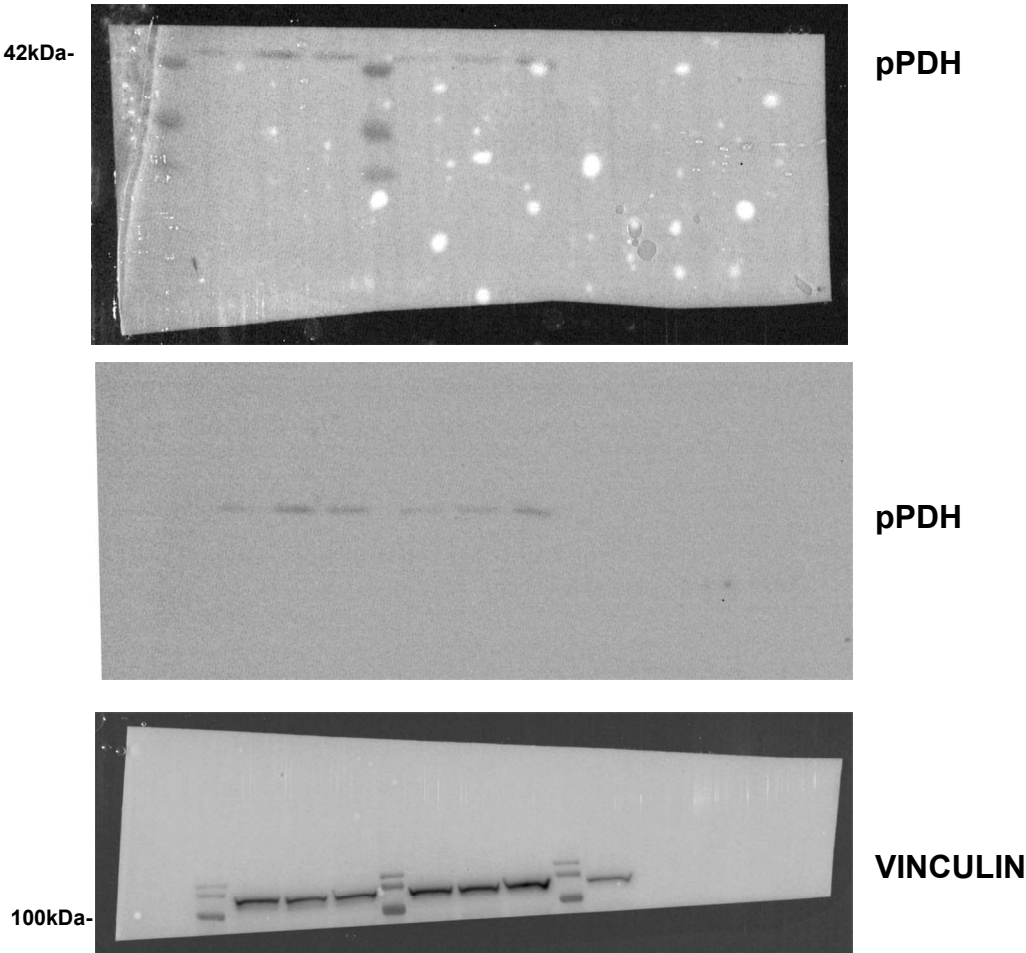

Supplement: Supplementary file 4 — Source Data for Expanded View and Appendix [file EMMM-12-e11217-s009.zip › EMM-2019-11217-V3-Original_Blots_Figure_EV2-sd.pdf]

**Fig. 1**

**F)**

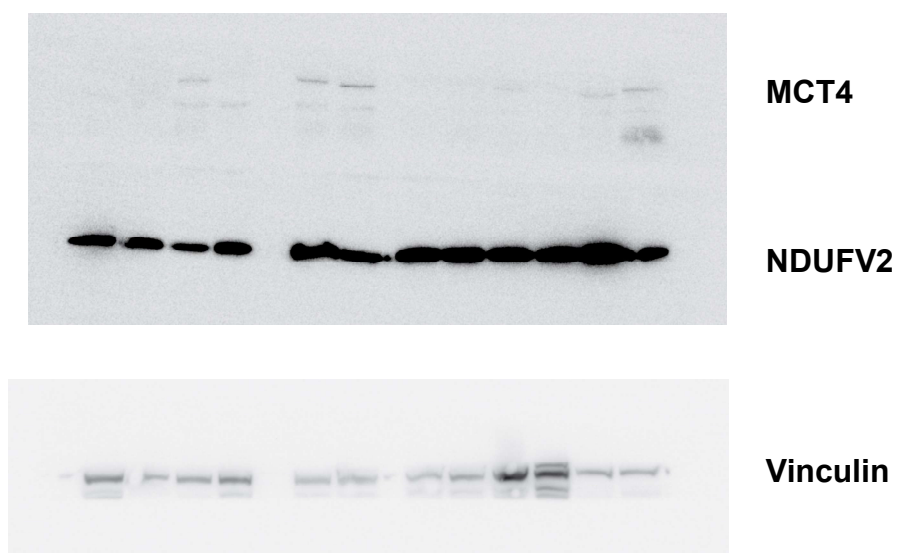

Supplement: Supplementary file 6 — Source Data for Figure 1 [file EMMM-12-e11217-s004.pdf]

Fig. 2

D)

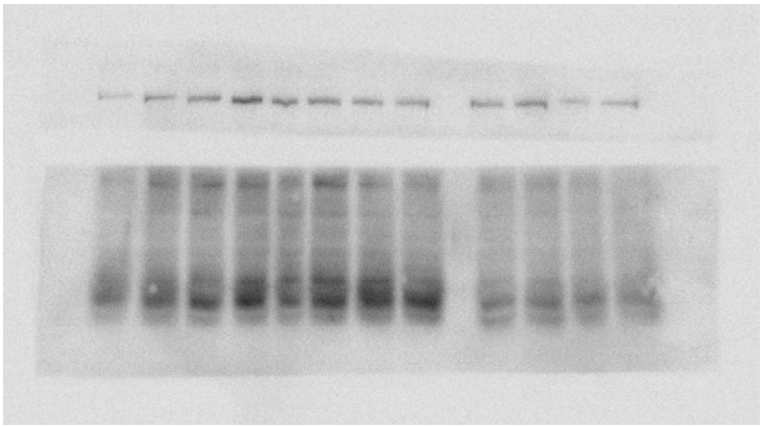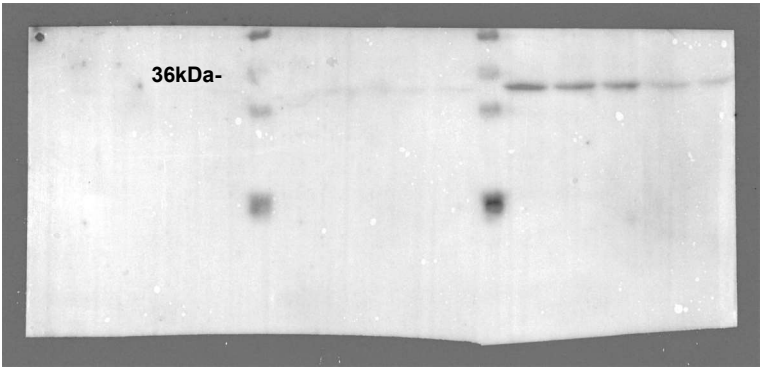

E)

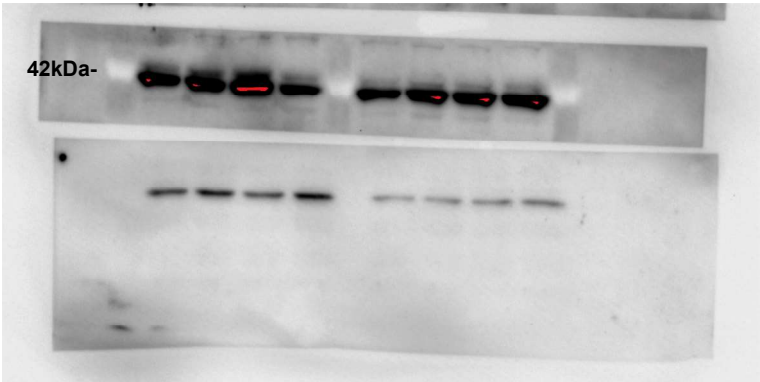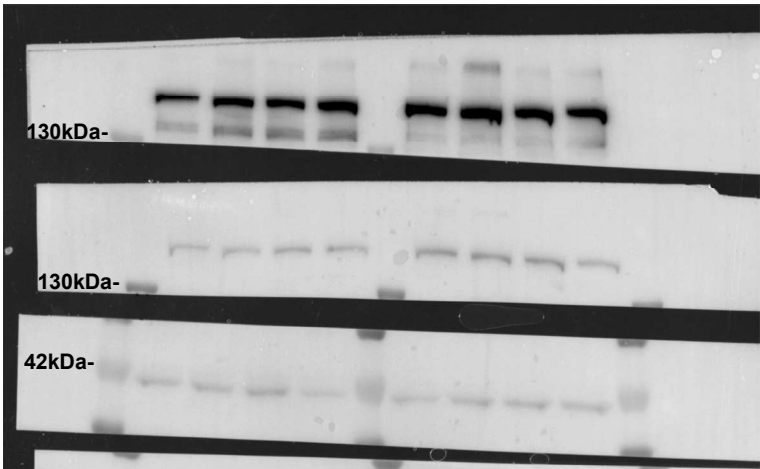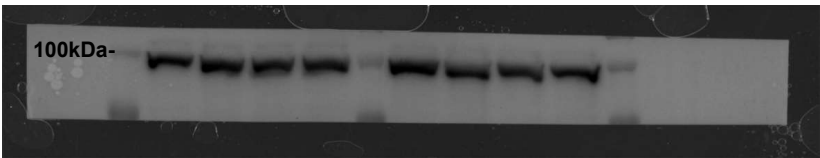

Supplement: Supplementary file 7 — Source Data for Figure 2 [file EMMM-12-e11217-s005.zip › EMM-2019-11217-V3-Original_Blots_Figure_2-sd.pdf]

Fig. 3

C) pH2A.X

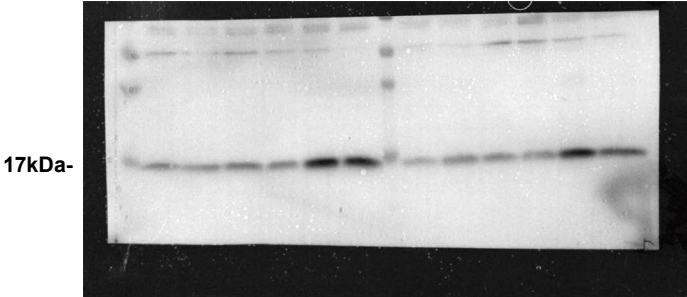

Tubulin

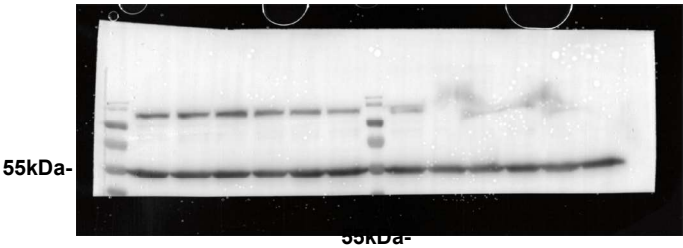

D) PARP1

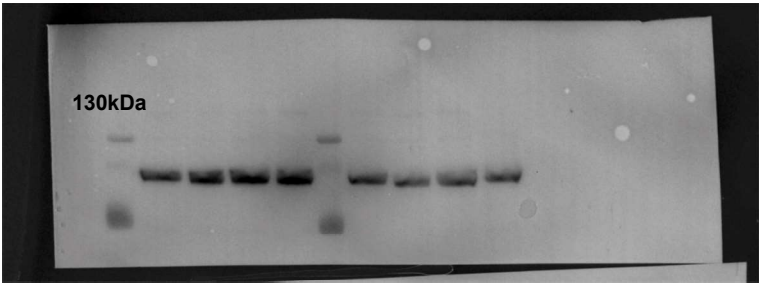

Tubulin

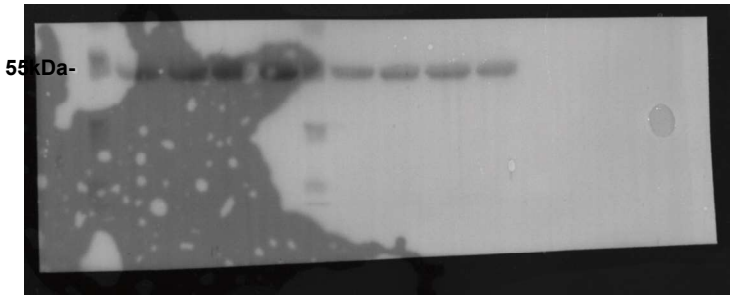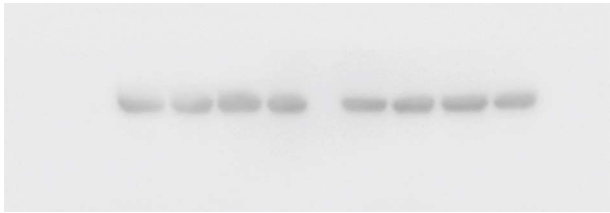

E) PAR

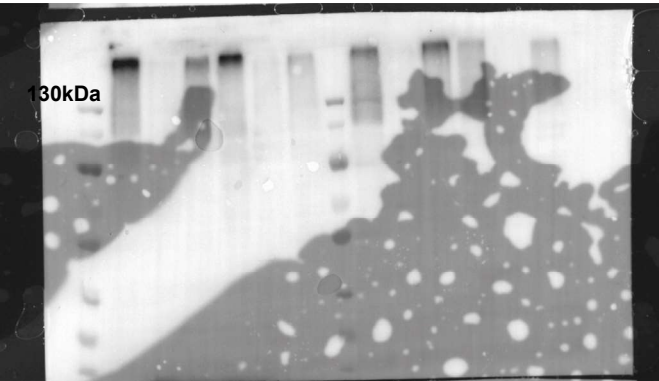

Tubulin

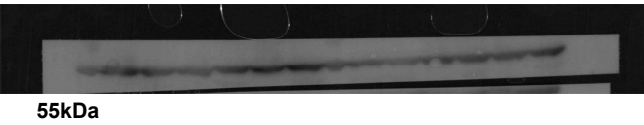

F) PAR

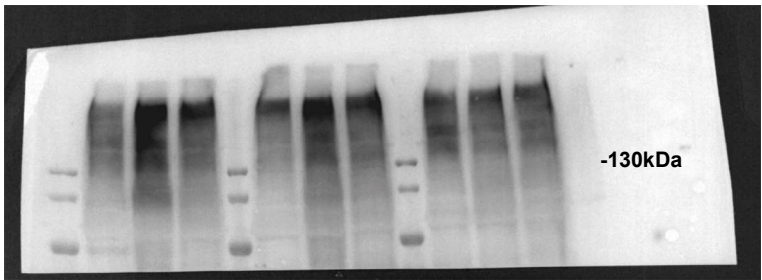

Tubulin

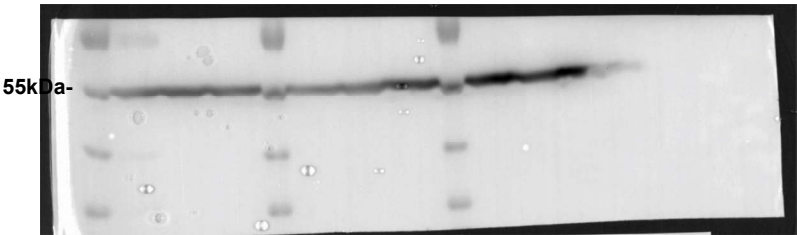

Supplement: Supplementary file 8 — Source Data for Figure 3 [file EMMM-12-e11217-s006.pdf]

**Fig. 4**

**E)**

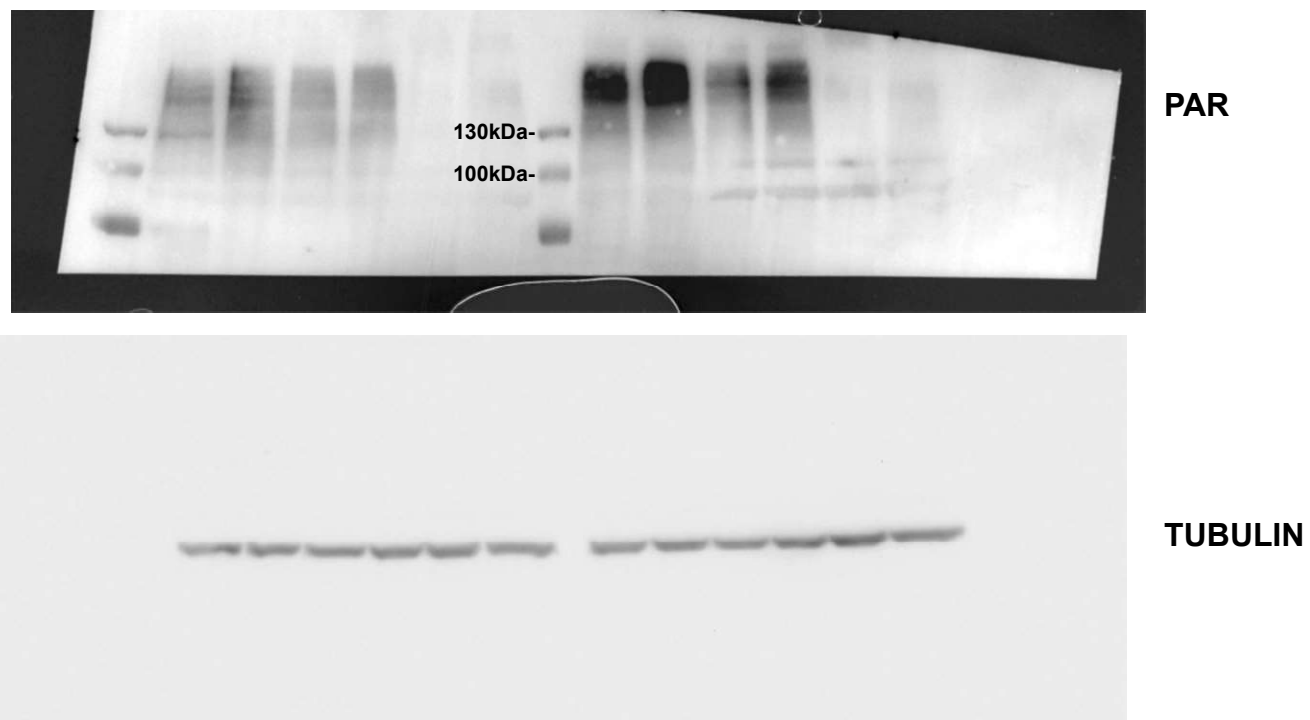

Supplement: Supplementary file 9 — Source Data for Figure 4 [file EMMM-12-e11217-s007.zip › EMM-2019-11217-V3-Original_Blots_Figure_4-sd.pdf]

Fig. 6

C)

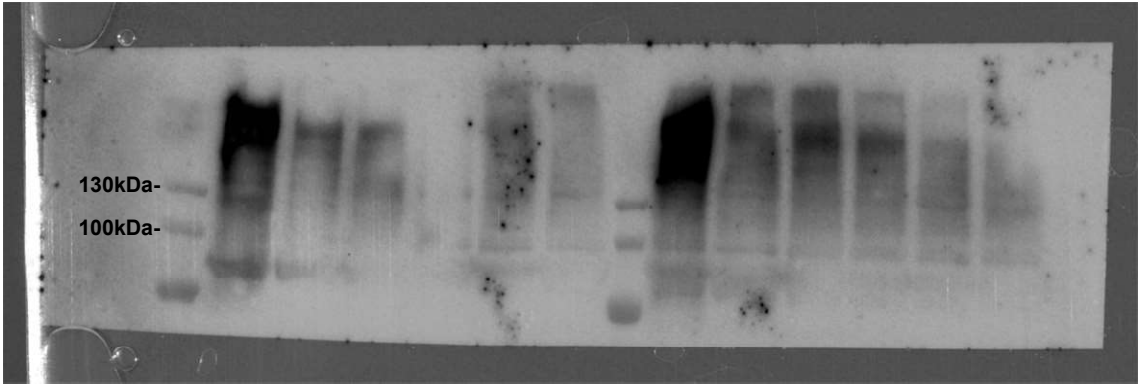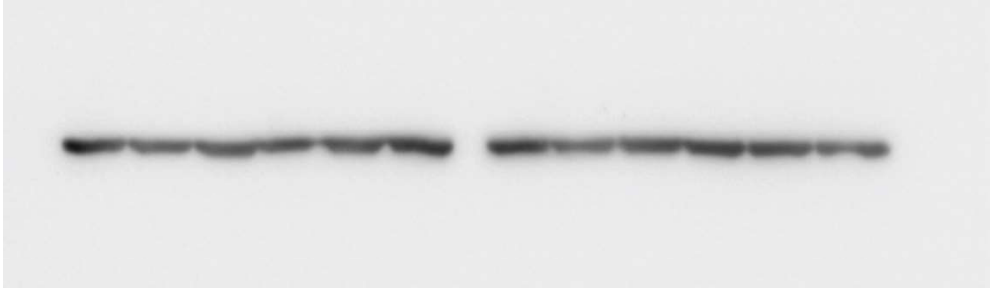

D)

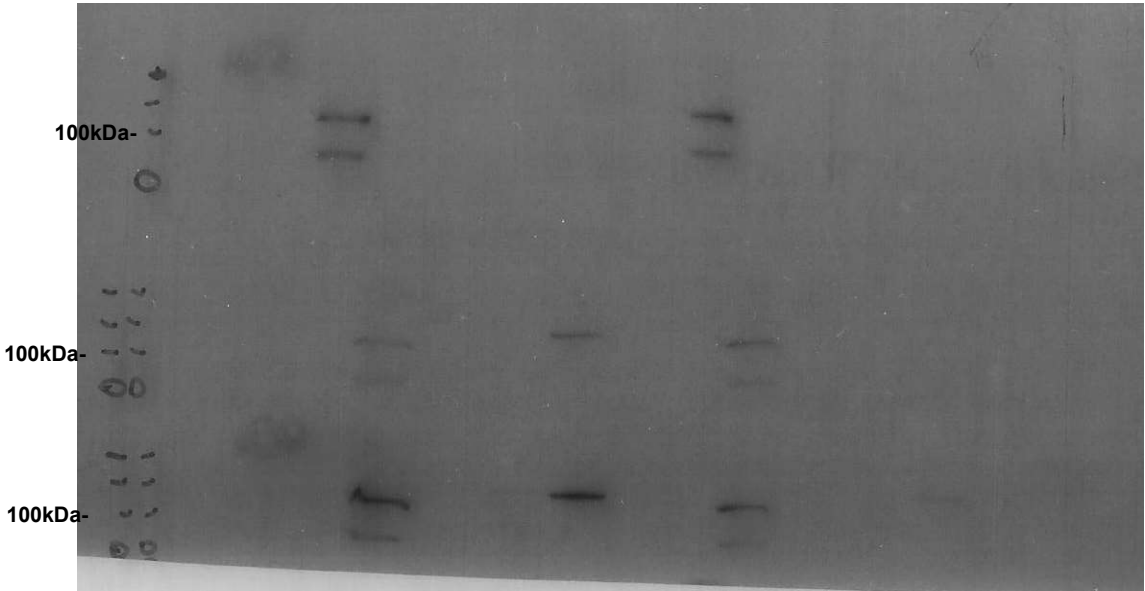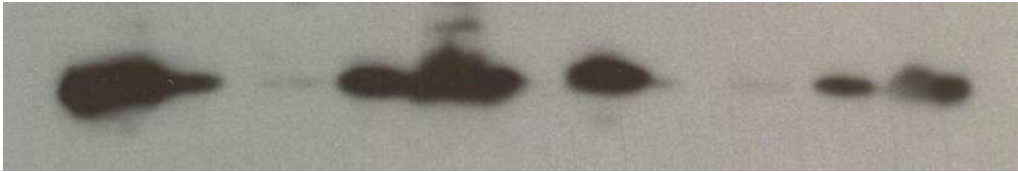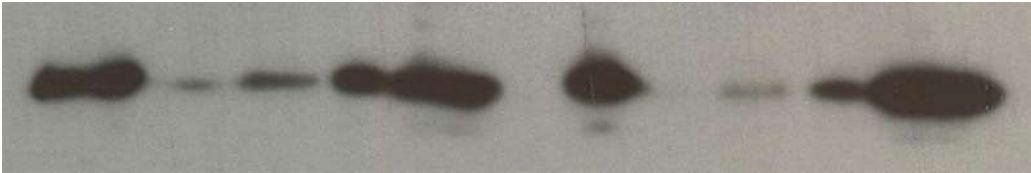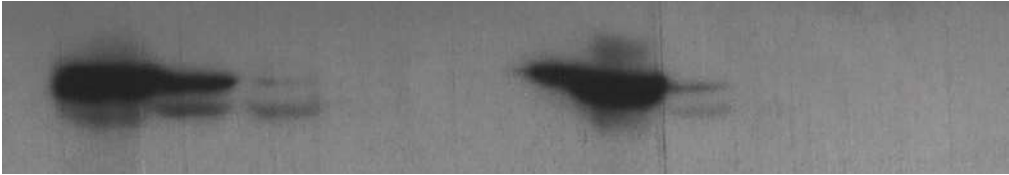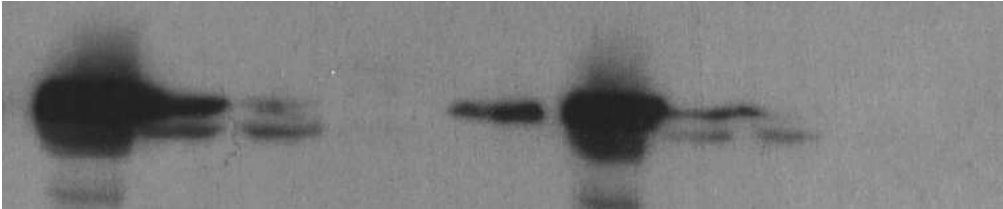

Supplement: Supplementary file 10 — Source Data for Figure 6 [file EMMM-12-e11217-s008.pdf]
