# Supplementary material for: Tumors defective in homologous recombination rely on oxidative metabolism: relevance to treatments with PARP inhibitors
Source: EMBO Mol Med. 2020 May 13;12(6):e11217. doi: 10.15252/emmm.201911217 (PMC7278557; doi:10.15252/emmm.201911217)

Appendix Figure S2

B)

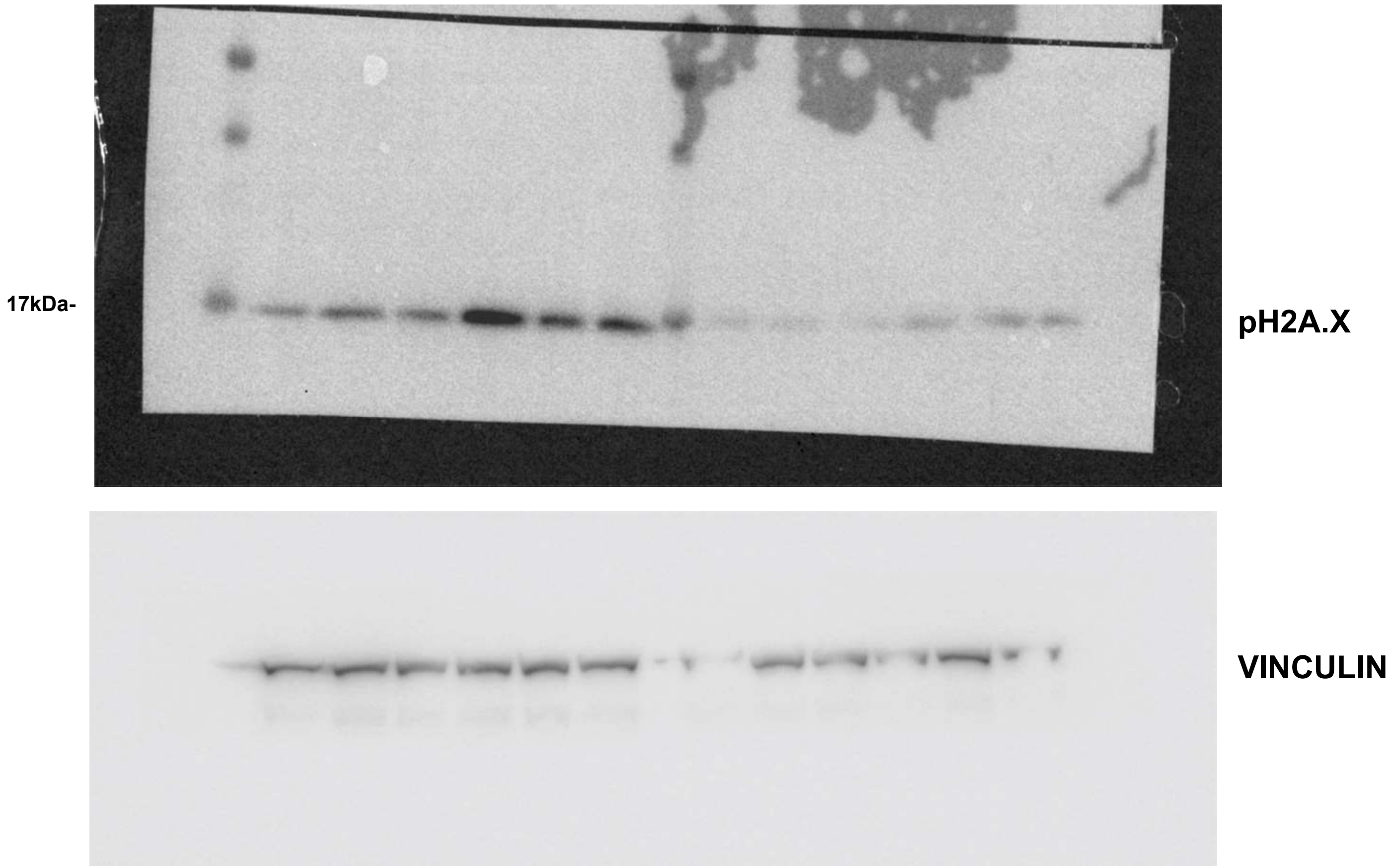

Supplement: Supplementary file 4 — Source Data for Expanded View and Appendix [file EMMM-12-e11217-s009.zip › Appendix_Figures_Source_Data.zip!/EMM-2019-11217-V3-Original_Blots_Appendix_Figure_S2-sd.pdf]
